# Synthesis, LSD1 Inhibitory Activity, and LSD1 Binding Model of Optically Pure Lysine-PCPA Conjugates

**DOI:** 10.5936/csbj.201402002

**Published:** 2014-02-15

**Authors:** Yukihiro Itoh, Daisuke Ogasawara, Yosuke Ota, Tamio Mizukami, Takayoshi Suzuki

**Affiliations:** a Graduate School of Medical Science, Kyoto Prefectural University of Medicine, 13 Taishogun Nishitakatsukasa-cho, Kita-ku, Kyoto 603-8334, Japan; b Graduate School of Bio-Science, Nagahama Institute of Bio-Science Technology, 1226 Tamura-cho, Nagahama, Shiga 526-0829, Japan; c PRESTO, Japan Science and Technology Agency (JST), 4-1-8 Honcho, Kawaguchi, Saitama 332-0012, Japan

**Keywords:** epigenetics, histone demethylase, lysine-specific demethylase, protein-targeted drug delivery, ligand-protein interaction, drug discovery

## Abstract

Compounds that inhibit the catalytic function of lysine-specific demethylase 1 (LSD1) are interesting as therapeutic agents. Recently, we identified three lysine-phenylcyclopropylamine conjugates, NCD18, NCD25, and NCD41, which are potent LSD1 inactivators. However, in our previous study, because we tested those compounds as mixtures of (1*S*,2*R*)- and (1*R*,2*S*)-disubstituted cyclopropane rings, the relationship between the stereochemistry of the cyclopropane ring and their biological activity remained unknown. In this work, we synthesized optically active compounds of NCD18, NCD25, and NCD41 and evaluated their LSD1 inhibitory activities. In enzyme assays, the LSD1 inhibitory activities of (1*R*,2*S*)-NCD18 and (1*R*,2*S*)-NCD25 were approximately eleven and four times more potent than those of the corresponding (1*S*,2*R*)-isomers, respectively. On the other hand, (1*S*,2*R*)-NCD41 was four times more potent than (1*R*,2*S*)-NCD41. Binding simulation with LSD1 indicated that the aromatic rings of the compounds and the amino group of the cyclopropylamine were important for the interaction with LSD1, and that the stereochemistry of the 1,2-disubstituted cyclopropane ring affected the position of the aromatic rings and the hydrogen bond formation of the amino group in the LSD1 catalytic site. These findings are expected to contribute to the further development of LSD1 inactivators.

## Introduction

Lysine-specific demethylase 1 (LSD1) catalyzes the demethylation of mono- and dimethylated Lys4 of histone H3 through flavin adenine dinucleotide (FAD)-dependent enzymatic oxidation [1–3]. LSD1 regulates epigenetic gene expression that does not involve changes in the underlying DNA sequence, and plays key roles in such biological functions as embryonic development and homeostasis [4]. In particular, LSD1 is regarded as a drug target for various cancers, including leukemia and colon cancer [4–7]. In addition, it has been reported that LSD1 is associated with the latent infection of α-herpesvirus and globin disorder [8, 9]. However, the detailed biological/physiological roles of LSD1 and the association between LSD1 and oncogenic transformation remain unclear. Therefore, compounds that inhibit the catalytic activity of LSD1 are interesting as chemical tools for studying the functions of LSD1 and as candidate therapeutic agents targeting LSD1.

**Chart 1:**
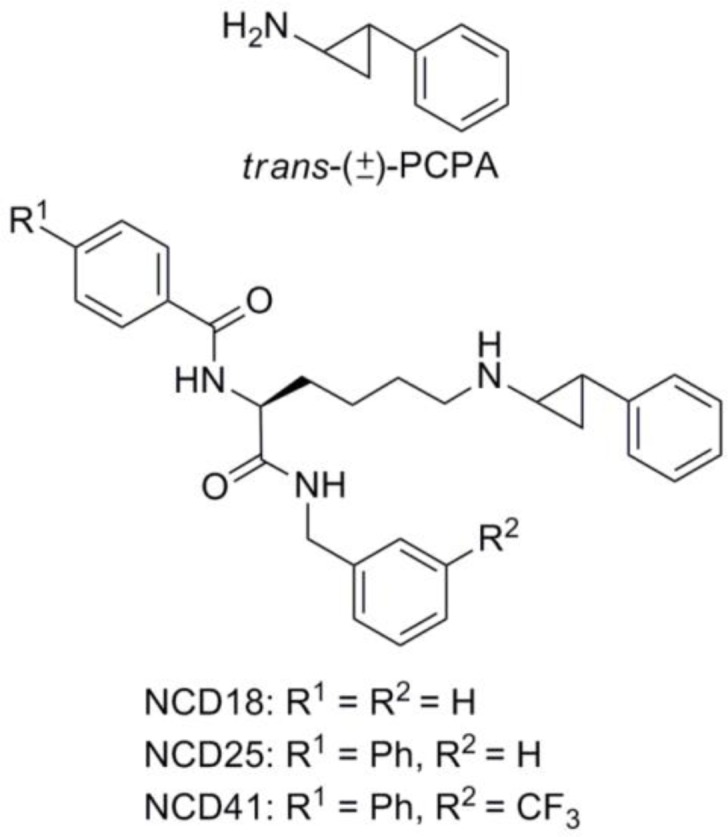
Structures of PCPA, NCD18, NCD25, and NCD41.

*trans*-2-Phenylcyclopropylamine (PCPA/Tranylcypromine, Chart 1) is one of the best-studied LSD1 inhibitors and has contributed immensely to the understanding of the biology of LSD1 [6, 10–13]. PCPA inhibits LSD1 irreversibly by forming a PCPA-FAD adduct through an enzymatic reaction with LSD1. However, PCPA has some drawbacks, including insufficient inhibitory potency and inadequate selectivity for LSD1 [14], because it was originally found as an inhibitor of monoamine oxidases (MAOs), which are also FAD-dependent enzymes. To this end, many PCPA derivatives have been developed to overcome those drawbacks [15–20].

Recently, we discovered a novel series of LSD1 inactivators (NCD series compounds, Chart 1) based on the idea of PCPA delivery to the LSD1 active site [21]. The NCD series compounds designed by conjugating PCPA with a lysine moiety, an LSD1-recognition structure, inactivated LSD1 strongly and selectively. However, they were synthesized by coupling a lysine moiety with racemic *trans*-PCPA and therefore, optically active NCD compounds were not evaluated for their LSD1 inhibitory activities. Some LSD1 research groups have investigated the difference in LSD1 inhibitory activity between the two enantiomers of PCPA and its derivatives [14, 22, 23]. Following those results, we decided to examine the relationship between the stereochemistry and the LSD1 inhibitory activities of the NCD series compounds. Here we report the synthesis, the LSD1 inhibitory activity, and the binding simulation of the optically active NCD compounds, with focus on the stereochemistry of the PCPA moiety.

## Experimental Procedures

### General procedure

Melting points were determined using a Yanagimoto micro melting point apparatus, a Büchi 545 melting point apparatus or a Yanaco micro melting point apparatus and were left uncorrected. Proton nuclear magnetic resonance spectra (^1^H NMR) and carbon nuclear magnetic resonance spectra (^13^C NMR) were recorded on a BRUKER AVANCE 300 spectrometer in the indicated solvent. Chemical shifts (δ) were reported in parts per million relative to the internal standard, tetramethylsilane. Elemental analysis was performed with a Yanaco CHN CORDER NT-5 analyzer, and all values were within ±0.4% of the calculated values. Fast atom bombardment (FAB) mass spectra were recorded on a JEOL JMS-SX102A mass spectrometer. HPLC analysis and Preparative HPLC were performed on an ODS-3 (150 mm x φ4.6 mm, GL Science or Cosmosil) and an Inertsil ODS-3 (250 mm x φ20 mm, GL Science or Cosmosil), respectively. The HPLC system was composed of a pump (HITACHI, L-6050 intelligent pump) and a detector (HITACHI, L-4000 UV detector) Elution of HPLC chromatogram in synthetic procedure was done with a linear gradient (eluent, 0 min (30% MeCN/0.1% TFA aq.) – 2 min (30% MeCN/0.1% TFA aq.) – 20 min (70% MeCN/0.1% TFA aq.) – 30 min (70% MeCN/0.1% TFA aq.).; flow rate = 1.0 mL/min). The detection wavelength was 254 nm. Optical rotation of optically active compounds was measured using a HORIBA Scientific, SEPA-300 polarimeter. Reagents and solvents were purchased from Aldrich, Merck, Nacalai Tesque, Tokyo Kasei Kogyo, Wako Pure Chemical Industries, Kishida Kagaku and Kanto Kagaku, and used without purification. Flash column chromatography was performed using Silica Gel 60 (particle size 0.046–0.063 mm) supplied by Merck.

### Preparation of optically active LSD1 inactivators

(1*S*,2*R*)-PCPA and (1*R*,2*S*)-PCPA were separated from commercially available *trans*-(±) PCPA by diastereomeric salt (salt **1** or **2**) formation method using optically pure tartaric acid [24]. The absolute stereostructures were confirmed by specific rotation [14, 24] and ^1^H NMR analysis using a chiral shift reagent, (18-crown-6)-2,3,11,12-tetracarboxylic acid [25, 26].

Optically pure PCPA (6.8–7.0 eq.) and potassium carbonate (K_2_CO_3_) (1.0 eq.) were added to a solution of corresponding mesylate compound **3** (1.0 eq.) in dimethylformamide (DMF) (0.2–0.3 mL) and the resulting mixture was stirred at 40–50 °C for 15–22 hours. The reaction mixture was diluted with dichloromethane and washed with aqueous saturated sodium hydrogen carbonate, brine and dried over sodium sulfate. Filtration and purification by silica gel flash column chromatography (chloroform/methanol = 100/0 to 50/1) gave optically active NCD18, NCD25 and NCD41 as crude solids. (1*S*,2*R*)-NCD18 was purified by preparative HPLC to give an optically active form as trifluoroacetic acid (TFA) salt. On the other hand, (1*R*,2*S*)-NCD18, (1*R*,2*S*)-NCD25, (1*S*,2*R*)-NCD25, (1*R*,2*S*)-NCD41 and (1*S*,2*R*)-NCD41 were neutralized with 4N hydrogen chloride in ethyl acetate and recrystallized from iso-propanol/diethyl ether (NCD18) or dichloromethane/diethyl ether (NCD25 and NCD41) to give optically active forms as hydrochloride salts. The detail characterization of each substance is described as below.

(1*R*,2*S*)-NCD18 hydrochloride salts: colorless amorphous (18.6 mg, 17%); ^1^H NMR (CD_3_OD, 300MHz, δ; ppm) 7.89–7.86 (2H, m), 7.55 (1H, t, *J* = 7.20 Hz), 7.46 (2H, t, *J* = 7.05 Hz), 7.33–7.14 (10H, m), 4.63–4.58 (1H, m), 4.42–4.39 (2H, m), 3.16 (2H, t, *J* = 7.65 Hz), 2.98–2.91 (1H, m), 2.50 (1H, sep, *J* = 3.39 Hz), 2.04–1.74 (4H, m), 1.64–1.32 (4H, m); ^13^C NMR (CD_3_OD, 75MHz, δ; ppm) 174.2, 170.4, 139.9, 139.4, 135.2, 133.0, 129.8, 129.6, 129.6, 128.6, 128.6, 128.3, 128.1, 127.5, 55.2, 44.2, 39.1, 32.5, 26.7, 24.2, 22.5, 13.4; HRMS (FAB) calcd for C_29_H_34_O_2_N_3_^+^, 456.2651, found, 456.2654; HPLC *t*_R_ = 18.72 min, purity 100%.

(1*S*,2*R*)-NCD18 hydrochloride salts: colorless crystals (74.9 mg, 55%); ^1^H NMR (CD_3_OD, 300MHz, δ; ppm) 7.89–7.85 (2H, m), 7.55 (1H, t, *J* = 7.35 Hz), 7.46 (2H, t, *J* = 7.35 Hz), 7.33–7.14 (10H, m), 4.63–4.58 (1H, m), 4.40 (2H, d, *J* = 1.86 Hz), 3.19–3.12 (2H, m), 2.93 (1H, quin, *J* = 2.93 Hz), 2.45 (1H, sep, *J* = 3.39 Hz), 2.04-1.72 (4H, m), 1.63–1.32 (4H, m); ^13^C NMR (CD_3_OD, 75MHz, δ; ppm) 174.2, 170.4, 139.8, 139.3, 135.2, 133.0, 129.8, 129.6, 129.6, 128.6, 128.5, 128.3, 128.1, 127.4, 55.1, 44.2, 39.1, 32.5, 26.7, 24.2, 22.5, 13.4; HRMS (FAB) calcd for C_29_H_34_O_2_N_3_^+^, 456.2651, found, 456.2625; HPLC *t*_R_ = 18.94 min, purity 96%.

(1*R*,2*S*)-NCD25 hydrochloride salt: colorless crystals (41.0 mg, 38%); m.p. 128–131 °C; ^1^H NMR (CD_3_OD, 300MHz, δ; ppm) 7.96 (2H, d, *J* = 8.70 Hz), 7.73 (2H, d, *J* = 8.40 Hz), 7.67 (2H, d, *J* = 7.20 Hz), 7.47 (2H, t, *J* = 7.20 Hz), 7.38 (1H, t, *J* = 7.20 Hz), 7.34–7.15 (10H, m), 4.66–4.61 (1H, m), 4.42 (2H, s), 3.22–3.13 (2H, m), 2.94 (1H, quin, *J* = 3.90 Hz), 2.47 (1H, sep, *J* = 3.40 Hz), 2.02–1.72 (4H, m), 1.65–1.33 (4H, m); ^13^C NMR (CD_3_OD, 75MHz, δ; ppm) 174.2, 170.1, 146.1, 141.2, 139.9, 139.3, 133.8, 130.1, 129.8, 129.6, 129.3, 128.6, 128.3, 128.2, 128.1, 127.5, 55.2, 44.2, 39.1, 32.6, 26.8, 24.2, 22.6, 13.5; HRMS (FAB) calcd for C_35_H_38_O_2_N_3_^+^, 532.2964, found, 532.2969; HPLC *t*_R_ = 12.54 min, purity 96.5%.

(1*S*,2*R*)-NCD25 hydrochloride salt: colorless crystals (42.4 mg, 39%); m.p. 121–123 °C; ^1^H NMR (CD_3_OD, 300MHz, δ; ppm) 7.96 (2H, d, *J* = 8.70 Hz), 7.73 (2H, d, *J* = 8.70 Hz), 7.67 (2H, d, *J* = 6.90 Hz), 7.47 (2H, t, *J* = 7.35 Hz), 7.38 (1H, t, *J* = 7.35 Hz), 7.33–7.14 (10H, m), 4.66–4.61 (1H, m), 4.42 (2H, s), 3.22–3.12 (2H, m), 2.95 (1H, quin, *J* = 3.98 Hz), 2.46 (1H, sep, *J* = 3.35 Hz), 2.04–1.72 (4H, m), 1.64–1.33 (4H, m); ^13^C NMR (CD_3_OD, 75MHz, δ; ppm) 174.2, 170.1, 146.1, 141.2, 139.9, 139.4, 133.8, 130.1, 129.8, 129.6, 129.3, 128.6, 128.3, 128.2, 128.1, 127.5, 55.1, 44.2, 39.2, 32.6, 26.8, 24.2, 22.6, 13.5; HRMS (FAB) calcd for C_35_H_38_O_2_N_3_^+^, 532.2964, found, 532.2968; Anal. calcd. for C_35_H_38_Cl N_3_O_2_·4/5H_2_O: C, 72.16; H, 6.85; N, 7.21, found: C, 72.03; H, 6.54; N, 7.14; HPLC *t*_R_ = 12.51 min, purity 95.9%.

(1*R*, 2*S*)-NCD41 hydrochloride salt: colorless crystals (62.6 mg, 62%); m.p. 166–170 °C; ^1^H NMR (CD_3_OD, 300MHz, δ; ppm) 7.97 (2H, d, *J* = 8.70 Hz), 7.75–7.15 (16H, m), 4.66–4.61 (1H, m), 4.49 (2H, s), 3.21–3.14 (2H, m), 2.95 (1H, quin, *J* = 3.90 Hz), 2.46 (1H, sep, *J* = 3.40 Hz), 2.05–1.73 (4H, m), 1.65–1.33 (4H, m); ^13^C NMR (CD_3_OD, 75MHz, δ; ppm) 174.5, 170.1, 146.1, 141.5, 141.2, 139.3, 133.7, 132.3, 130.4, 130.1, 129.8, 129.2, 129.2, 128.1, 128.1, 127.4, 125.2, 125.1, 125.0, 125.0, 55.2, 43.6, 39.1, 32.4, 26.7, 24.2, 22.5, 13.4.; HRMS (FAB) calcd. for C_36_H_37_F_3_O_2_N_3_^+^, 600.2838, found, 600.2841; Anal. Calcd. for C_36_H_37_ClF_3_ N_3_O_2_: C, 67.97; H, 5.86; N, 6.61. Found: C, 67.61; H, 5.64; N, 6.39.; HPLC *t*_R_ = 14.55 min, purity 97.5%.

(1*S*, 2*R*)-NCD41 hydrochloride salt: colorless crystals (62.4 mg, 68%); m.p. 134–136 °C; ^1^H NMR (CD_3_OD, 300MHz, δ; ppm) 7.97 (2H, d, *J* = 8.49 Hz), 7.75–7.15 (16H, m), 4.66–4.61 (1H, m), 4.49 (2H, s), 3.22–3.14 (2H, m), 2.95 (1H, quin, *J* = 3.90 Hz), 2.46 (1H, sep, *J* = 3.40 Hz), 2.04–1.74 (4H, m), 1.64–1.33 (4H, m); ^13^C NMR (CD_3_OD, 75MHz, δ; ppm) 174.5, 170.1, 146.1, 141.5, 141.3, 139.3, 133.8, 132.3, 130.4, 130.1, 129.8, 129.3, 129.2, 128.2, 128.1, 127.5, 125.2, 125.1, 125.0, 125.0, 55.2, 43.7, 39.1, 32.4, 26.8, 24.3, 22.6, 13.5; HRMS (FAB) calcd. for C_36_H_37_F_3_O_2_N_3_^+^, 600.2838, found, 600.2843; Anal. Calcd. for C_36_H_37_ClF_3_ N_3_O_2_: C, 67.97; H, 5.86; N, 6.61. Found: C, 67.89; H, 5.64; N, 6.61.; HPLC *t*_R_ = 14.54 min, purity 98.1%.

### LSD1 inhibition assay

The LSD1 inhibition assay was carried out according to the method reported in ref. 21.

### Binding simulation

Docking was performed using Molegro Virtual Docker 5.0 software. Coordinates of LSD1 completed with FAD-*N*-propargyl lysine peptide adduct were taken from the Brookhaven Protein Data Bank (PDB code 2UXN). Water molecules, cofactors and a peptide substrate were removed, and FAD was converted to a cofactor. The structure of NCD18, NCD25 and NCD41 bound to LSD1 was constructed by MolDock, which is based on a heuristic search algorithm that combines differential evolution with a cavity prediction algorithm. The docking parameters were as follow: Grid Resolution: 0.30, Max iterations: 1500, Population size: 50, Energy threshold: 100.00, Simplex evolution: 300 (Max steps) and 1.00 (Neighbour distance factor), Search space: (X, Y, Z) = (65.64, 47.97, 35.60) with radius 10, distance constraints (for N atom of cyclopropylamine of NCD18, NCD25, NCD41): constraint center (X, Y, Z) = (64.51, 54.11, 34.83) with hard constraint between minimum 0 to maximum 2.0.

## Results and Discussion

### Chemistry

**Chart 2:**
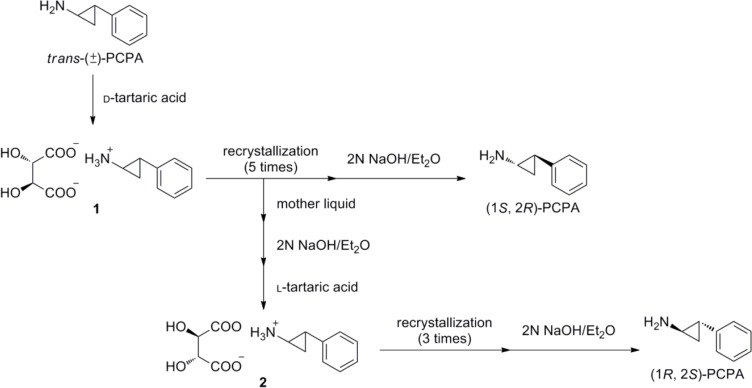
Preparation of optically pure *trans*-PCPA.

**Chart 3:**
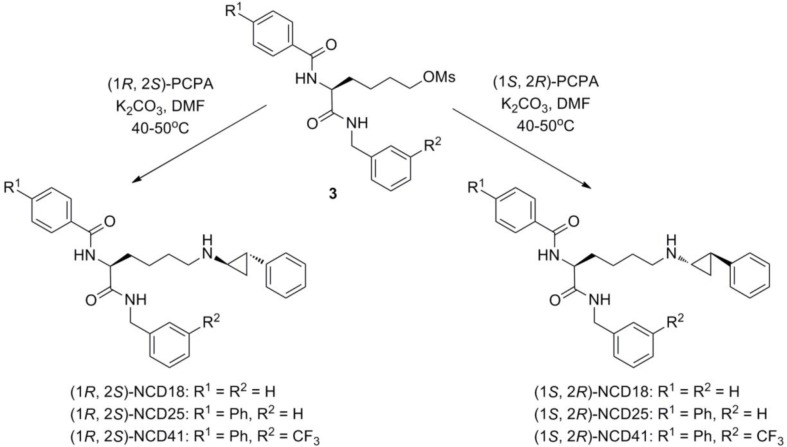
Synthesis of optically pure LSD1 inactivators.

Individual stereoisomers were synthesized as shown in Charts 2 and 3. First, *trans*-(±) PCPA was resolved into d-tartaric acid salt **1** and the salt was recrystallized. Then, simple deprotonation with sodium hydroxide liberated free (1*S*,2*R*)-PCPA. On the other hand, (1*R*,2*S*)-PCPA was collected from the mother liquid by extraction with ether, salt formation (salt **2**) with l-tartaric acid, recrystallization of the d-tartaric acid salt, and extraction with ether in sequential order. Finally, each enantiomer was reacted with the corresponding mesylate compound **3** [21] in the presence of potassium carbonate to give optically pure NCD18, NCD25, and NCD41 in 17–68% yield.

### Enzyme assays

We examined the LSD inhibitory activities of optically active NCD18, NCD25, and NCD41 by means of a horseradish peroxidase-coupled assay [27] (Table 1). As expected, the diastereomers of NCD18, NCD25, and NCD41 inhibited LSD1 more strongly than PCPA. Importantly, there were differences in LSD1 inhibitory activity between (1*R*,2*S*)- and (1*S*,2*R*)-isomers. The IC_50_ values of (1*R*,2*S*)-NCD18, (1*R*,2*S*)-NCD25, and (1*S*,2*R*)-NCD41 were lower than those of (1*S*,2*R*)-NCD18, (1*S*,2*R*)-NCD25, and (1*R*,2*S*)-NCD41, respectively. The results revealed that the (1*R*,2*S*)-isomers are superior to the (1*S*,2*R*)-isomers with respect to LSD1 inhibition by NCD18 and NCD25. On the other hand, the (1*S*,2*R*)-isomer of NCD41 was more potent than its (1*R*,2*S*)-isomer. Although we could not conclude which isomer is more potent, we found that the LSD1 inhibitory activities of the (1*S*,2*R*)-isomer and the (1*R*,2*S*)-isomer are dependent on the structure of the R group in the NCD series compounds (Chart 3).

**Table 1:** Inhibitory activities of LSD1 inactivators.

Inactivator	IC_50_ (µM)			Ratio

	(1*RS*,2*RS*)	(1*R*,2*S*)	(1*S*,2*R*)	(1*S*,2*R*)/(1*R*,2*S*)
PCPA	31^a^	ND^b^	ND^b^	-
NCD18	0.30^a^	0.13	1.4	11
NCD25	0.48^a^	0.16	0.62	3.9
NCD41	0.58^a^	1.4	0.31	0.22

a Taken from the literature (ref 21).

b ND = No data.

### Binding simulation

**Figure 1:**
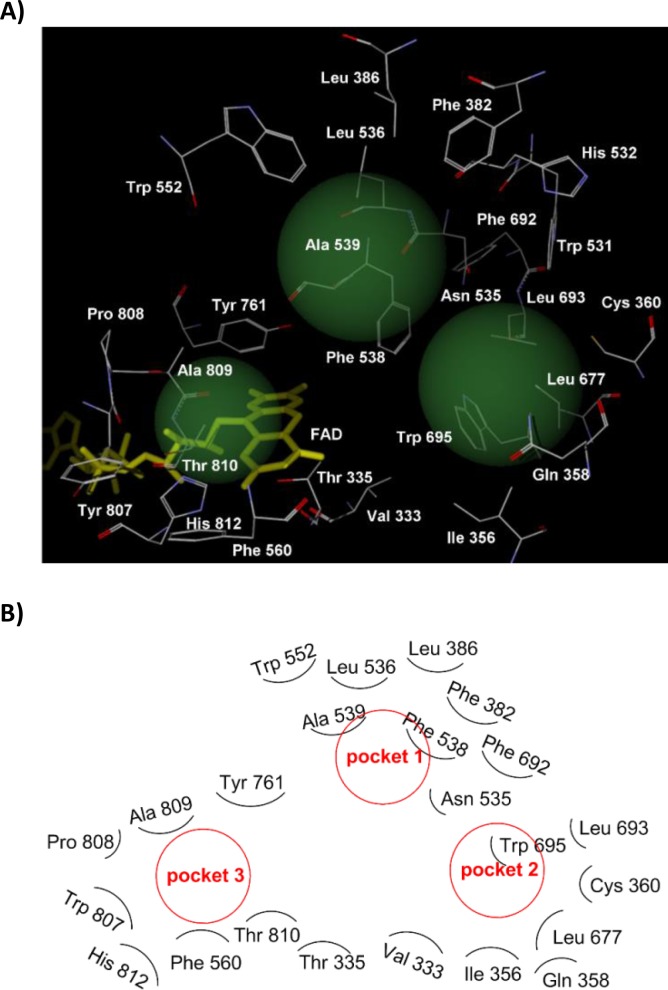
(**A**) View of the three hydrophobic pockets in the LSD1 catalytic core. Each pocket is drawn as a green sphere. Pocket 1 is formed by Phe 382, Leu 386, Leu 536, Phe 538, Ala 539, Trp 552, and Phe 692. Pocket 2 is formed by Val 333, Ile 356, Leu 677, Leu 693, and Trp 695. Pocket 3 is formed by Phe 560, Tyr 807, Pro 808, Ala 809, Thr 810 (methyl group), and His 812. (**B**) Schematic diagram of Figure 1A.

To verify the relationship between the stereochemistry and the LSD1 inhibitory activity of the NCD series compounds, we performed binding simulation of the compounds to LSD1 by using Molegro Virtual Docker 5.0 software. The simulation was performed based on the reported X-ray structure of LSD1 [28] and under the condition that the cyclopropylamine group of NCD18, NCD25, and NCD41 was fixed to the position where it could react with FAD. The results of the simulation indicated that the interaction with amino acid residues in the three hydrophobic pockets (pockets 1–3, Figure 1) and the formation of one hydrogen bond are important for the LSD1 inhibitory activity of optically pure LSD1 inactivators as discussed in more detail below.

**Figure 2:**
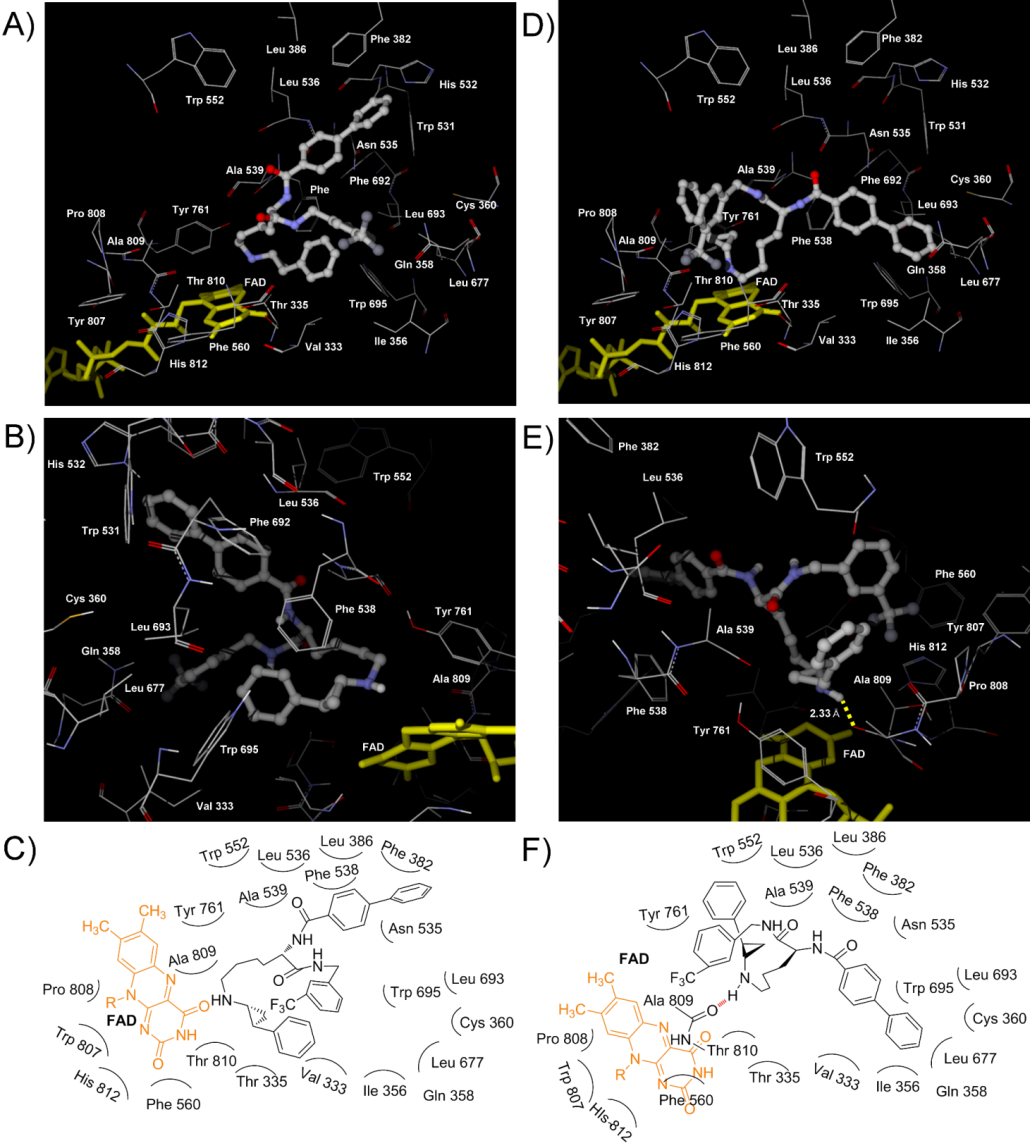
Views of NCD18 docked to LSD1 catalytic core. (A) and (B) Views of the binding conformation of (1*R*,2*S*)-NCD18 (ball and stick). The yellow broken line indicates a hydrogen bond and the value indicates distance between the H atom of the amino group of cyclopropylamine and the O atom of the side chain of Tyr 761. (C) Schematic diagram of binding of (1*R*,2*S*)-NCD18. (D) and (E) Views of the binding conformation of (1*S*,2*R*)-NCD18 (ball and stick). The yellow broken line indicates a hydrogen bond and the value indicates distance between the H atom of the amino group of cyclopropylamine and the O atom of the side chain of Tyr 761. (F) Schematic diagram of binding of (1*S*,2*R*)-NCD18.

First, we simulated the binding mode of NCD18. In the lowest energy complex of (1*R*,2*S*)-NCD18 with LSD1, as described in our previous report [21], the benzoyl group and the benzyl group of (1*R*,2*S*)-NCD18 could interact with hydrophobic amino acid residues in two of the three pockets (Figure 2A and 2C). The benzoyl group of (1*R*,2*S*)-NCD18 was positioned in pocket 1 formed by Phe 382, Leu 386, Leu 536, Phe 538, Ala 539, Trp 552, and Phe 692, and the benzyl group was positioned in pocket 2 formed by Val 333, Ile 356, Leu 677, Leu 693, and Trp 695. In addition, (1*R*,2*S*)-NCD18 could form a hydrogen bond with the oxygen atom of Tyr 761 (Figure 2B and 2C). On the other hand, (1*S*,2*R*)-NCD18 could not interact with any amino acid residues in the three hydrophobic pockets (Figure 2D–F), although it could form a hydrogen bond with Tyr 761 (Figure 2E). Those interactions with amino acid residues in the hydrophobic pockets may be the reason why (1*R*,2*S*)-NCD18 is more potent than its (1*S*,2*R*)-isomer.

**Figure 3:**
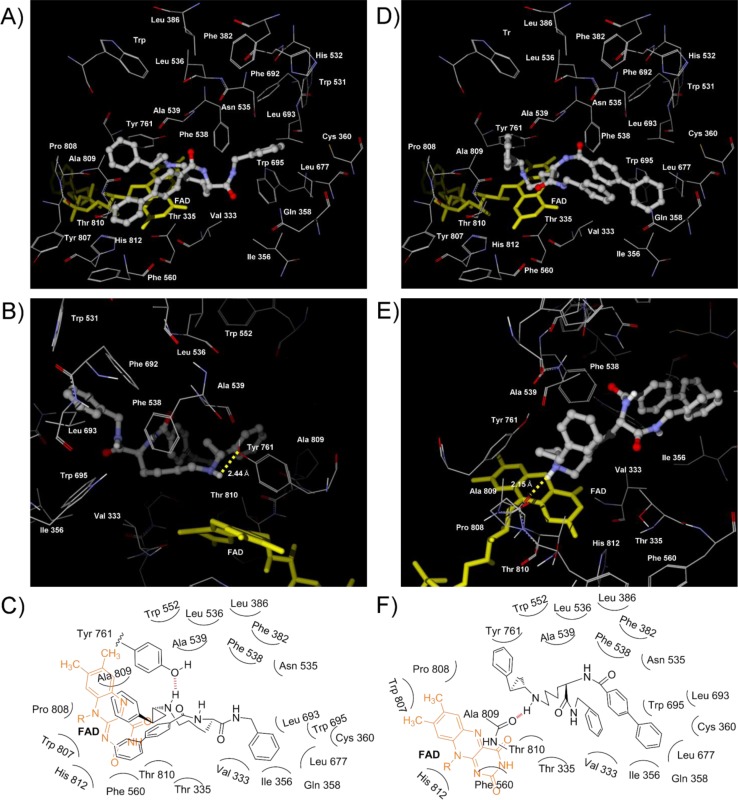
View of NCD25 docked to LSD1 catalytic core. (A) and (B) Views of the binding conformation of (1*R*,2*S*)-NCD25 (ball and stick). The yellow broken line indicates a hydrogen bond and the value indicates distance between the H atom of the amino group of cyclopropylamine and the O atom of the side chain of Tyr 761. (C) Schematic diagram of binding of (1*R*,2*S*)-NCD25. (D) and (E) Views of the binding conformation of (1*S*,2*R*)-NCD25 (ball and stick). The yellow broken line indicates a hydrogen bond and the value indicates distance between the H atom of the amino group of cyclopropylamine and the carbonyl oxygen of Ala 809. (F) Schematic diagram of binding of (1*S*,2*R*)-NCD25.

Next, the modeling of NCD25 binding with LSD1 was conducted. As shown in Figure 3A and 3C, the binding model of NCD25 was different from that of NCD18, i.e., the biphenyl group of (1*R*,2*S*)-NCD25 could interact with pocket 3 formed by Phe 560, Tyr 807, Pro 808, Ala 809, Thr 810 (methyl group), and His 812, although the benzyl group could interact with the hydrophobic amino acid residues in pocket 2 as in the case of (1*R*,2*S*)-NCD18 (Figure 2A and 2C). In the case of (1*S*,2*R*)-NCD25, the biphenyl group was positioned in pocket 2, but the benzyl group did not interact with any of the three pockets (Figure 3D and 3F). (1*R*,2*S*)-NCD25 and (1*S*,2*R*)-NCD25 could interact with the oxygen atom of Tyr 761 and the carbonyl oxygen of Ala 809 via a hydrogen bond (Figure 3B and 3E), respectively. Taken together, (1*R*,2*S*)-NCD25 could interact with two hydrophobic pockets whereas (1*S*,2*R*)-NCD25 could interact with only one hydrophobic pocket. This may be the reason why the LSD1 inhibitory activity of (1*R*,2*S*)-NCD25 is superior to that of (1*S*,2*R*)-NCD25.

**Figure 4:**
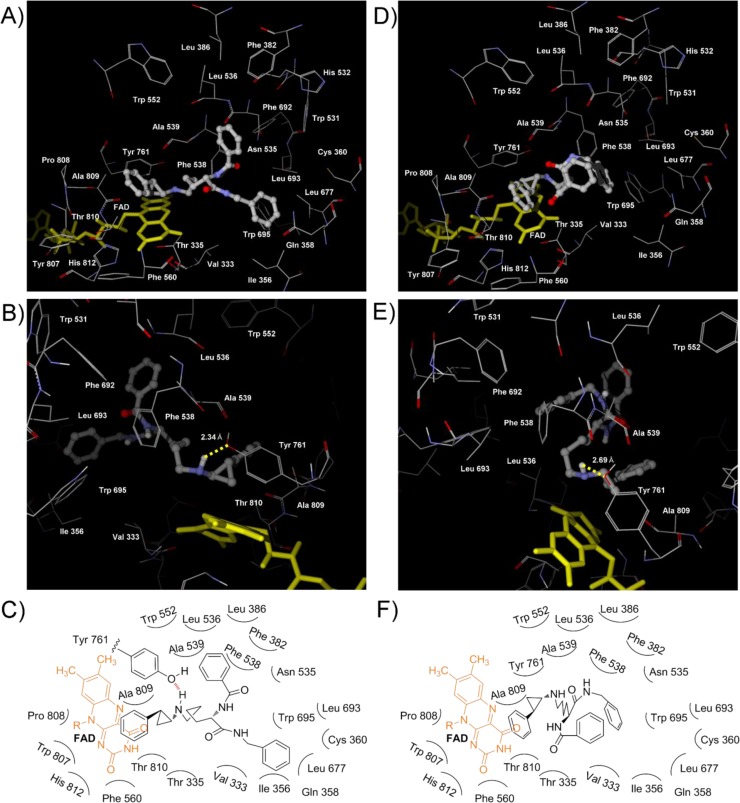
View of NCD41 docked to LSD1 catalytic core. (A) and (B) Views of the binding conformation of (1*R*,2*S*)-NCD41 (ball and stick). (C) Schematic diagram of binding of (1*R*,2*S*)-NCD41. (D) and (E) Views of the binding conformation of (1*S*,2*R*)-NCD41 (ball and stick). The yellow dotted line indicates a hydrogen bond and the value indicates distance between the H atom of the amino group of cyclopropylamine and the carbonyl oxygen of Ala 809. (F) Schematic diagram of binding of (1*S*,2*R*)-NCD41.

Finally, the modeling of NCD41 binding with LSD1 was carried out as in the case of NCD18 and NCD25. Although the biphenyl group of (1*R*,2*S*)-NCD41 could interact with the hydrophobic amino acid residues in pocket 1, (1*R*,2*S*)-NCD41 could not form any hydrogen bonds (Figure 4A and 4B). However, as in the case of (1*R*,2*S*)-NCD25 (Figure 3A and 3C), the biphenyl group and the trifluoromethylbenzyl group of (1*S*,2*R*)-NCD41 could interact with the amino acid residues in pocket 2 and pocket 3, respectively (Figure 4D and 4F). In addition, (1*S*,2*R*)-NCD41 could interact with the carbonyl oxygen of Ala 809 via a hydrogen bond (Figure 4E). The experimental results that (1*S*,2*R*)-NCD41 is more potent than its (1*R*,2*S*)-isomer could be explained by the calculated results.

The difference between NCD25 and NCD41 is the presence of *m*-trifluoromethyl group at the benzyl group (Chart 1). The introduction of this substituent to (1*R*,2*S*)-NCD25 could cause the sterical hinderance between the trifluoromethyl group and Leu 677 or Leu 693 in pocket 2 (Figure 3A and 3C). Therefore, *m*-trifluoromethylated benzyl group of (1*R*,2*S*)-NCD41 could not be positioned in pocket 2 Instead, the *m*-trifluoromethylated benzyl group of (1*R*,2*S*)-NCD41 was located in pocket 3 (Figure 4D and 4F) which is too large to appropriately accommodate the unsubstituted benzyl group of (1*R*,2*S*)-NCD25. This may be the reason why the inhibitory activity of (1*R*,2*S*)-NCD41 is superior to that of (1*R*,2*S*)-NCD25.

The interaction of each isomer with amino acid residues in the hydrophobic pockets and the hydrogen bond formation are summarized as follows: (i) (1*R*,2*S*)-NCD18, (1*R*,2*S*)-NCD25, and (1*S*,2*R*)-NCD41 could interact with amino acid residues in two hydrophobic pockets and form one hydrogen bond, (ii) (1*S*,2*R*)-NCD25 could interact with amino acid residues in only one hydrophobic pocket and form one hydrogen bond, (iii) (1*S*,2*R*)-NCD18 could form one hydrogen bond, and (iv) (1*R*,2*S*)-NCD41 could interact with amino acid residues in only one hydrophobic pocket. Considering the relationship between the stereochemistry and the LSD1 inhibitory activity of the NCD series compounds, compounds that could interact with amino acid residues in two pockets and form a hydrogen bond showed high LSD1 inhibitory activities. In other words, the stereochemistry of the PCPA moiety could affect LSD1 inhibitory activity through the interaction of the two aromatic groups with hydrophobic amino acid residues and the hydrogen bond formation.

## Conclusion

In conclusion, we synthesized optically active (1*R*,2*S*)-isomers and (1*S*,2*R*)-isomers of NCD18, NCD25, and NCD41, and evaluated their LSD1 inhibitory activities in enzyme assays. The (1*R*,2*S*)-isomers of NCD18 and NCD25 were more potent than their (1*S*,2*R*)-isomers. On the other hand, the (1*S*,2*R*)-isomer of NCD41 was more potent than its (1*R*,2*S*)-isomer. In addition, the binding simulation indicated that the potent NCD series compounds can interact with amino acid residues in the two hydrophobic pockets and form a hydrogen bond in the LSD1 active site, and that the stereochemistry of PCPA affects LSD1 inhibitory activity. These findings will be useful for the further development of PCPA-based LSD1 inhibitors and LSD1-targeted therapy.

## References

[1] Shi Y, Lan F, Matson C, Mulligan P, Whetstine JR, et al. (2004) Histone demethylation mediated by the nuclear amine oxidase homolog LSD1. Cell 119: 941–953 15620353 10.1016/j.cell.2004.12.012

[2] Culhane JC, Cole PA (2007) LSD1 and the chemistry of histone demethylation. Curr Opin Chem Biol 11: 561–568 17851108 10.1016/j.cbpa.2007.07.014 PMC2112775

[3] Ruthenburg AJ, Li H, Patel DJ, Allis CD. (2007) Multivalent engagement of chromatin modifications by linked binding modules. Nat Rev Mol Cell Biol 8: 983–994 18037899 10.1038/nrm2298 PMC4690530

[4] Shi Y (2007) Histone lysine demethylases: emerging roles in development, physiology and disease. Nat Rev Genet 8: 829–833 17909537 10.1038/nrg2218

[5] Scoumanne A, Chen X (2007) The lysine-specific demethylase 1 is required for cell proliferation in both p53-dependent and -independent manners. J Biol Chem 282: 15471–15475 17409384 10.1074/jbc.M701023200

[6] Schenk T, Chen WC, Gollner S, Howell L, Jin L, et al. (2012) Inhibition of the LSD1 (KDM1A) demethylase reactivates the *all*-trans-retinoic acid differentiation pathway in acute myeloid leukemia. Nat Med 18: 605–611 22406747 10.1038/nm.2661 PMC3539284

[7] Ding J, Zhang ZM, Xia Y, Liao GQ, Pan Y, et al. (2013) LSD1-mediated epigenetic modification contributes to proliferation and metastasis of colon cancer. Br J Cancer 109: 994–1003 23900215 10.1038/bjc.2013.364 PMC3749561

[8] Liang Y, Vogel JL, Narayanan A, Peng H, Kristie TM (2009) Inhibition of the histone demethylase LSD1 blocks ⊠-herpesvirus lytic replication and reactivation from latency. Nat Med 15: 1312–1317 19855399 10.1038/nm.2051 PMC2783573

[9] Shi L, Cui S, Engel JD, Tanabe O (2013) Lysine-specific demethylase 1 is a therapeutic target for fetal hemoglobin induction. Nat Med 19: 291–294 23416702 10.1038/nm.3101 PMC5512162

[10] Schmidt DMZ, McCafferty DG (2007) *trans*-2-Phenylcyclopropylamine is a mechanism-based inactivator of the histone demethylase LSD1. Biochemistry 46: 4408–4416 17367163 10.1021/bi0618621

[11] Yang M, Culhane JC, Szewczuk LM, Jalili P, Ball HL, et al. (2007) Structural basis for the inhibition of the LSD1 histone demethylase by the antidepressant *trans*-2-phenylcyclopropylamine. Biochemistry 46: 8058–8065 17569509 10.1021/bi700664y

[12] Lim S, Janzer A, Becker A, Zimmer A, Schüle R, et al. (2010) Lysine-specific demethylase 1 (LSD1) is highly expressed in ER-negative breast cancers and a biomarker predicting aggressive biology. Carcinogenesis 31: 512–520 20042638 10.1093/carcin/bgp324

[13] Schulte JH, Lim S, Schramm A, Friedrichs N, Koster J, et al. (2009) Lysine-specific demethylase 1 is strongly expressed in poorly differentiated neuroblastoma: implications for therapy. Cancer Res 69: 2065–2071 19223552 10.1158/0008-5472.CAN-08-1735

[14] Binda C, Valente S, Romanenghi M, Pilotto S, Cirilli R, et al. (2010) Biochemical, structural, and biological evaluation of tranylcypromine derivatives as inhibitors of histone demethylases LSD1 and LSD2. J Am Chem Soc 132: 6827–6833 20415477 10.1021/ja101557k

[15] Ueda R, Suzuki T, Mino K, Tsumoto H, Nakagawa H, et al. (2009) Identification of cell-active lysine specific demethylase 1-selective inhibitors. J Am Chem Soc 131: 17536–17537 19950987 10.1021/ja907055q

[16] Gooden DM, Schmidt DMZ, Pollock JA, Kabadi AM, McCafferty DG (2008) Facile synthesis of substituted *trans*-2-arylcyclopropylamine inhibitors of the human histone demethylase LSD1 and monoamine oxidases A and B. Bioorg Med Chem Lett 18: 3047–3051 18242989 10.1016/j.bmcl.2008.01.003 PMC2661354

[17] Mimasu S, Umezawa N, Sato S, Higuchi T, Umehara T, et al. (2010) Structurally designed trans-2-phenylcyclopropylamine derivatives potently inhibit histone demethylase LSD1/KDM1. Biochemistry 49: 6494–6503 20568732 10.1021/bi100299r

[18] Harris WJ, Huang X, Lynch JT, Spencer GJ, Hitchin JR, et al. (2012) The histone demethylase KDM1A sustains the oncogenic potential of MLL-AF9 leukemia stem cells. Cancer Cell 21: 473–487 22464800 10.1016/j.ccr.2012.03.014

[19] Suzuki T, Miyata N (2011) Lysine demethylases inhibitors. J Med Chem 54: 8236–8250 21955276 10.1021/jm201048w

[20] Itoh Y, Suzuki T, Miyata N (2013) Small-molecular modulators of cancer-associated epigenetic mechanisms. Mol Biosyst 9: 873–896 23511667 10.1039/c3mb25410k

[21] Ogasawara D, Itoh Y, Tsumoto H, Kakizawa T, Mino K, et al. (2013) Lysine-specific demethylase 1-selective inactivators: protein-targeted drug delivery mechanism. Angew Chem Int Ed Engl 52: 8620–8624 23824985 10.1002/anie.201303999

[22] Ogasawara D, Suzuki T, Mino K, Ueda R, Khan MN, et al. (2010) Synthesis and biological activity of optically active NCL-1, a lysine-specific demethylase 1 selective inhibitor. Bioorg Med Chem 19: 3702–3708 21227703 10.1016/j.bmc.2010.12.024

[23] Benelkebir H, Hodgkinson C, Duriez PJ, Hayden AL, Bulleid RA, et al. (2011) Enantioselective synthesis of tranylcypromine analogues as lysine demethylase (LSD1) inhibitors. Bioorg Med Chem 19: 3709–3716 21382717 10.1016/j.bmc.2011.02.017

[24] Malancona S, Altamura S, Filocamo G, Kinzel O, Hernando JIM, et al. (2011) Identification of MK-5710 ((8*aS*)-8a-methyl-1,3-dioxo-2-[(1*S*,2*R*)-2-phenylcyclopropyl]-*N*-(1-phenyl-1*H*-pyrazol-5-yl)hexahydroimid azo[1,5-a]pyrazine-7(1H)-carboxamide), a potent smoothened antagonist for use in Hedgehog pathway dependent malignancies, Part 1. Bioorg Med Chem Lett 21: 4422–4428 21737272 10.1016/j.bmcl.2011.06.024

[25] Wenzel TJ, Thurston JE (2000) 18-Crown-6)-2,3,11,12-Tetracarboxylic acid and its ytterbium(III) complex as chiral NMR discriminating agents. J Org Chem 65: 1243–1248 10814083 10.1021/jo9913154

[26] Lovely AE, Wenzel TJ (2006) Chiral NMR discrimination of piperidines and piperazines using (18-Crown-6)-2,3,11,12-tetracarboxylic acid. J Org Chem 71: 9178–9182 17109544 10.1021/jo061586w

[27] Forneris F, Binda C, Vanoni MA, Battaglioli E, Mattevi A (2005) Human histone demethylase LSD1 reads the histone code. J Biol Chem 280: 41360–41365 16223729 10.1074/jbc.M509549200

[28] Yang M, Culhane JC, Szewczuk LM, Gocke CB, Brautigam CA, et al. (2007) Structural basis of histone demethylation by LSD1 revealed by suicide inactivation. Nat Struct Mol Biol 14: 535–539 17529991 10.1038/nsmb1255

